# Phylogenetic context of Shiga toxin-producing *Escherichia coli* serotype O26:H11 in England

**DOI:** 10.1099/mgen.0.000551

**Published:** 2021-03-24

**Authors:** Timothy J. Dallman, David R. Greig, Saheer E. Gharbia, Claire Jenkins

**Affiliations:** ^1^​ National Infection Service, Public Health England, London, NW9 5EQ, UK; ^2^​ Division of Infection and Immunity, The Roslin Institute and Royal (Dick) School of Veterinary Studies, University of Edinburgh, Easter Bush, EH25 9RG, UK

**Keywords:** foodborne outbreaks, public health, Shiga toxin-producing *Escherichia coli*O26:H11, surveillance, whole genome sequencing

## Abstract

The increasing use of PCR for the detection of gastrointestinal pathogens in hospital laboratories in England has improved the detection of Shiga toxin-producing *Escherichia coli* (STEC), and the diagnosis of haemolytic uraemic syndrome (HUS). We aimed to analyse the microbiological characteristics and phylogenetic relationships of STEC O26:H11, clonal complex (CC) 29, in England to inform surveillance, and to assess the threat to public health. There were 502 STEC belonging to CC29 isolated between 2014 and 2019, of which 416 were from individual cases. The majority of isolates belonged to one of three major sequence types (STs), ST16 (*n*=37), ST21 (*n*=350) and ST29 (*n*=24). ST16 and ST29 were mainly isolated from cases reporting recent travel abroad. Within ST21, there were three main clades associated with domestic acquisition. All three domestic clades had Shiga toxin subtype gene (*stx*) profiles associated with causing severe clinical outcomes including STEC-HUS, specifically either *stx1a*, *stx2a* or *stx1a/stx2a*. Isolates from the same patient, same household or same outbreak with an established source for the most part fell within 5-SNP single linkage clusters. There were 19 5-SNP community clusters, of which six were travel-associated and one was an outbreak of 16 cases caused by the consumption of contaminated salad leaves. Of the remaining 12 clusters, 9/12 were either temporally or geographically related or both. Exposure to foodborne STEC O26:H11 ST21 capable of causing severe clinical outcomes, including STEC-HUS, is an emerging risk to public health in England. The lack of comprehensive surveillance of this STEC serotype is a concern, and there is a need to expand the implementation of methods capable of detecting STEC in local hospital settings.

## Data Summary

All FASTQ files were submitted to the National Centre for Biotechnology Information (NCBI). All data can be found under BioProject: PRJNA315192 – https://www.ncbi.nlm.nih.gov/bioproject/?term=PRJNA315192. Strain-specific details can be found in the Methods section under data deposition.

**Impact Statement**

*
Escherichia coli* serotype O26:H11 were a known cause of gastrointestinal disease in the UK prior to the emergence of Shiga toxin-producing *E. coli* (STEC) O157:H7 in the 1980s. However, surveillance strategies focused on STEC O157:H7 because it was associated with outbreaks of haemolytic uraemic syndrome (HUS). Here we show that over the last four decades, strains of STEC O26:H11 circulating in England have acquired the gene encoding Shiga toxin (*stx*) 2a, the Stx subtype associated with causing STEC-HUS. Despite limited surveillance of the non-O157 STEC serotypes in the UK, we provide evidence that STEC O26:H11 is causing outbreaks of foodborne disease, can be transmitted from person to person in household settings, and that children continue to shed STEC O26:H11 in their faeces for weeks after becoming asymptomatic. STEC O26:H11 is an emerging threat to public health in the UK, and the need for improved microbiological and epidemiological surveillance is highlighted.

## Introduction

Shiga toxin-producing *Escherichia coli* (STEC) belong to a pathogenic group of zoonotic *E. coli* that cause severe gastrointestinal (GI) symptoms in humans, mainly due to their ability to produce Shiga toxin (Stx) [1]. STEC serotype O157:H7 emerged in the UK in the early 1980s as a cause of outbreaks of haemolytic uraemic syndrome (HUS) in children [2]. HUS is a severe condition that is characterized by renal failure, and sometimes cardiac and/or neurological complications, and can be fatal [3]. Subsequent studies showed that the emergence of STEC serotype O26:H11, belonging to clonal complex (CC) 29, in the UK pre-dates that of STEC O157:H7 [4]. This serotype played an essential part in the early work on identifying the pathogenic mechanisms of STEC, specifically Stx [5, 6].

There are two types of Stx, Stx1 and Stx2, and a number of subtypes (Stx1a–1d and Stx2a–2g) and genes encoding these toxins are located on bacteriophages. Historically, the majority of strains of STEC O26:H11 in the UK and elsewhere had *stx1a* only [7, 8], but isolates containing *stx2a* have been increasingly reported in many countries [9–12]. There is evidence to show that strains that have *stx2a* or *stx2d* have a higher potential to cause HUS compared to strains that have *stx1a* only [7, 13]. Like STEC O157:H7, STEC O26:H11 also have genes encoding proteins involved in attaching to the gut mucosa, including the *E. coli* attaching and effacing gene (*eae*) located on the locus of enterocyte effacement [14].

STEC O26:H11 colonize the gut of ruminants, such as cattle, sheep and goats, and other animals including small mammals, birds and domestic pets may act as transient vectors [15, 16]. Transmission to humans occurs following the consumption of contaminated food or water, and direct contact with animals or their environment [17–19]. There is evidence of person-to-person spread in households and institutional settings, and the infectious dose is likely to be similar to that of STEC O157:H7 (10–100 organisms) [20, 21].

Due to the association of STEC O157:H7 with HUS in the UK, laboratory methods have focused on the use of media that were selective for this specific serotype, and surveillance of other STEC serotypes is limited [22]. Therefore, the true burden of human disease in the UK caused by STEC O26:H11 is unknown [23]. In countries adopting a more comprehensive approach to the detection of STEC using immuno-sorbent assays detecting the production of Stx or molecular assays targeting *stx*, the incidence of STEC O26:H11 is similar to that of STEC O157:H7 [10, 24–26]. Since 2013, an increasing number of hospital laboratories in England have implemented PCR for the detection of GI pathogens [23, 27]. This PCR detects the presence of *stx*, the defining characteristic of the STEC group, and therefore all STEC serotypes, including STEC O26:H11.

SNP typing derived from whole genome sequencing (WGS) data can be used to enhance the public health surveillance of STEC, including the detection and investigation of outbreaks [28]. Sequence similarity, based on hierarchical single linkage clustering of pairwise SNP distances of pathogen genomes, can infer the relatedness between isolates as the fewer SNPs identified between pairs of isolates, the less time since divergence from a common ancestor [29, 30]. As such, isolates with very similar genomes have an increased likelihood that they are transmitted via the same vehicle and/or from the same source population. Furthermore, WGS can be used to determine the virulence profile, specifically *stx* subtype and presence of *eae*, of each isolate [31, 32]. The aim of this study was to analyse the genome-derived *stx* subtype and phylogenetic relationships of STEC O26:H11 in England to inform surveillance, and to assess the threat to public health.

## Methods

### Bacterial strains

Between January 2014 and December 2019, there were 502 isolates of CC29 from 416 individual patients detected at the Gastrointestinal Bacterial Reference Unit (GBRU), at Public Health England (PHE), from faecal specimens that were PCR positive for *stx* and/or from patients with clinical symptoms of HUS. The faecal specimens were from hospital and community cases of GI disease that were submitted to the GBRU from local and regional hospital laboratories in England. Previously, Ogura *et al*. [8] provided a phylogenetic overview of the population structure of STEC O26:H11 belonging to CC29. In order to review sequences of STEC O26:H11 isolated from cases resident in England in the global phylogenetic context, 271 publicly available genomes of STEC O26:H11 from Ogura *et al.* [8] were downloaded and processed as described below.

### Whole genome sequencing

The isolates belonging to CC29 were inoculated into nutrient broth and propagated overnight at 37 °C. Genomic DNA from isolates of STEC O26:H11 was extracted on the QiaSymphony (Qiagen). The sequence library was prepared using the Nextera XT kit and sequenced on the HiSeq 2500 platform, using the fast protocol (Illumina), yielding paired-end reads of 100 bp in length. FASTQ reads were processed using Trimmomatic v0.27 to remove bases with a PHRED score of <30 from the leading and trailing ends, with reads <50 bp after quality trimming being discarded [33].

High-quality reads were mapped to the reference STEC O26:H11 strain, 11368 (GenBank accession NC_013361.1), using the Burrows–Wheeler Aligner – Maximum Exact Matching (BWA MEM, v0.7.2) [34]. The sequence alignment map output from BWA was sorted and indexed to produce a binary alignment map (BAM) using Samtools (v1.1) [35]. Genome Analysis Toolkit (GATK v2.6.5) [36] was then used to create a variant call format (VCF) file from each of the sorted BAMs, which were further parsed to extract only SNP positions of high quality [mapping quality (MQ) >30, depth (DP) >10, variant ratio >0.9] [30]. Hierarchical single linkage clustering was performed on the pairwise SNP difference between all isolates at descending distance thresholds (Δ250, Δ100, Δ50, Δ25, Δ10, Δ5, Δ0) [30]. The result of the clustering is an SNP profile, or SNP address, that is used to describe the population structure based on clonal group membership, as indicated by the number at each level of the seven-number SNP address. Visualization of distributions of pairwise SNP distances and statistical detection of outliers was analysed in Python using plotly (https://plotly.com/) and numpy (https://numpy.org/) respectively. Sunburst representations of SNP clustering was also produced using plotly within Python. Phylogenetic trees were constructed using RAxML v8.2.8 [37].

Serotypes were derived from the genome data using the GeneFinder tool, based on the Serotypefinder database [38] and the best match to each of the O and H determinants was reported, as described by Chattaway *et al*. [31]. Sequence type (ST) assignment was performed using the Metric Orientated Sequence Typer (MOST), available from https://github.com/phe-bioinformatics/MOST [39]. Stx subtyping was performed as previously described [32].

### Data availability

The sequences for all isolates in this study were stored in the National Center for Biotechnology Information (NCBI) Short Read Archive (SRA) BioProject: PRJNA315192.

### Data collection

Microbiological typing data, including serotype, sequence type and SNP type or SNP address, and patient demographic data including sex, age, residential area and recent travel were stored in an in-house integrated molecular national surveillance database. Travel-associated cases were defined as those reporting recent foreign travel to any country outside the UK 7 days prior to the onset of symptoms, based on information from laboratory reports.

### Case definitions

Household: a case who shared the same household as another case.

Outbreak: a case belonging to a cluster of cases where the source of the outbreak was determined.

Cluster: a case with an isolate belonging to the same 5- or 10-SNP single linkage cluster as an isolate from another case.

Sporadic: a case with an isolate that did not belong to a 5-SNP single linkage cluster as an isolate from another case.

## Results

### Phylogenetic relationships and *stx* profiles of isolates belonging to CC29

In this study, 411 of the 416 isolates from individual cases belonging to CC29 belonged to three major sequence types, ST16 (*n*=37), ST21 (*n*=350) and ST29 (*n*=24). Of the 350 isolates in ST21, 333/350 (95.1 %) were serotype O26:H11. Other serotypes belonging to ST21 included O69:H11 (*n*=6), O118:H16 (*n*=5), O71:H11 (*n*=3), O151:H16 (*n*=2) and O123:H11 (*n*=1). All but one of the ST16 isolates were serotype O111:H8 (36/37, 97.3 %), and ST29 comprised mostly serotypes O26:H11 (11/24, 45.8 %) and O177:H11 (7/24, 29.2 %). The majority of the isolates belonging to ST16 and ST29 had *stx1a* only, 33/37 (89.2 %) and 18/24 (75.0 %) respectively. Of the 350 isolates belonging to ST21, 207/350 (59.3 %) had *stx1a*, 66/350 (18.8 %) had *stx2a*, 58/350 (16.6 %) isolates had *stx1a/stx2a* and one isolate had *stx2d*. All isolates were positive for the gene encoding intimin (*eae*) (Supplementary material).

**Fig. 1.**
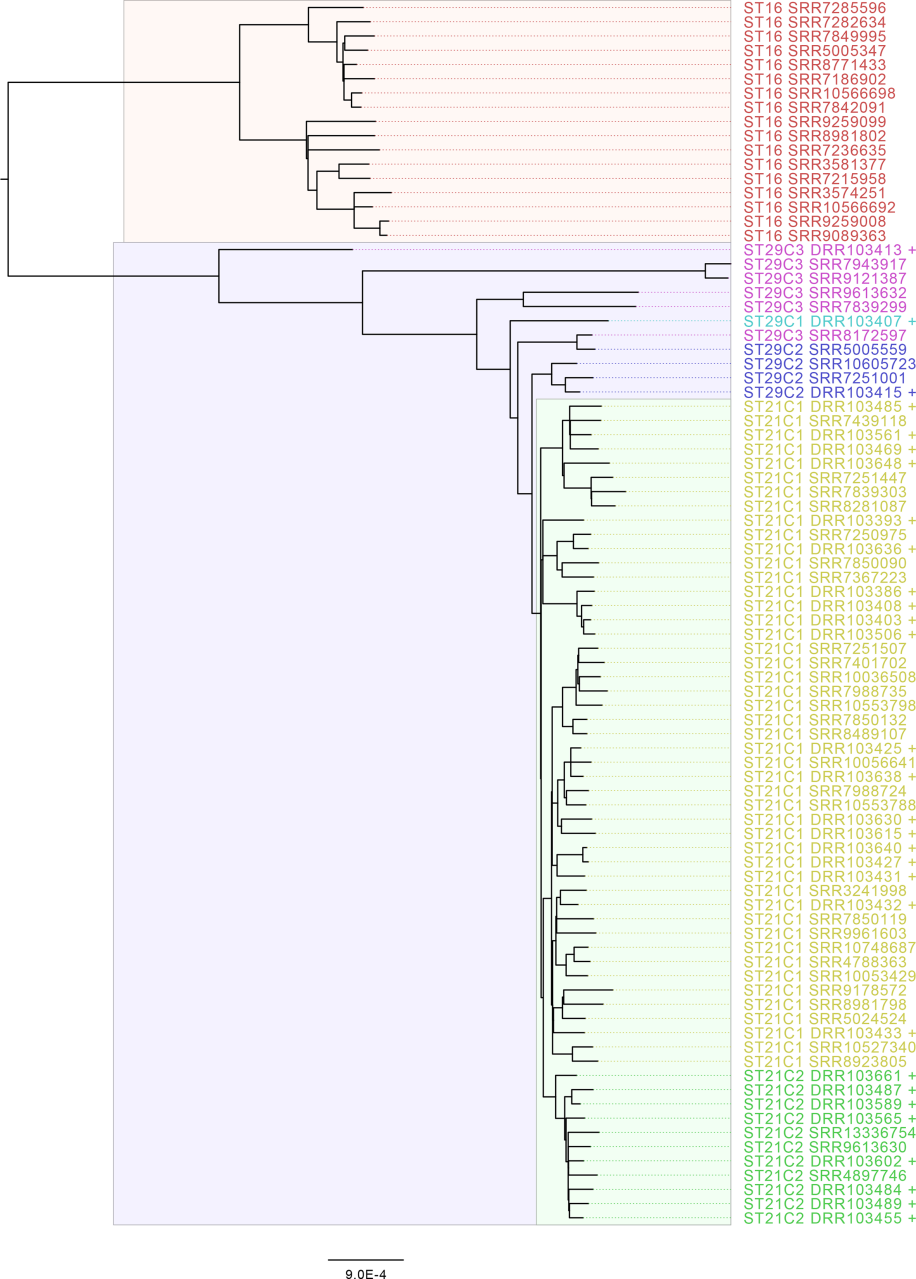
Mid-rooted phylogeny of *E. coli* CC21 encompassing representative sequences from each t:250 single linkage cluster from this study and Ogura *et al*. Sequences from Ogura *et al.* are labelled +. Clades are coloured by ST and ST clonal complex as defined in Ogura *et al*. [8]. Scale bars represent the number of substitutions per site.

**Fig. 2.**
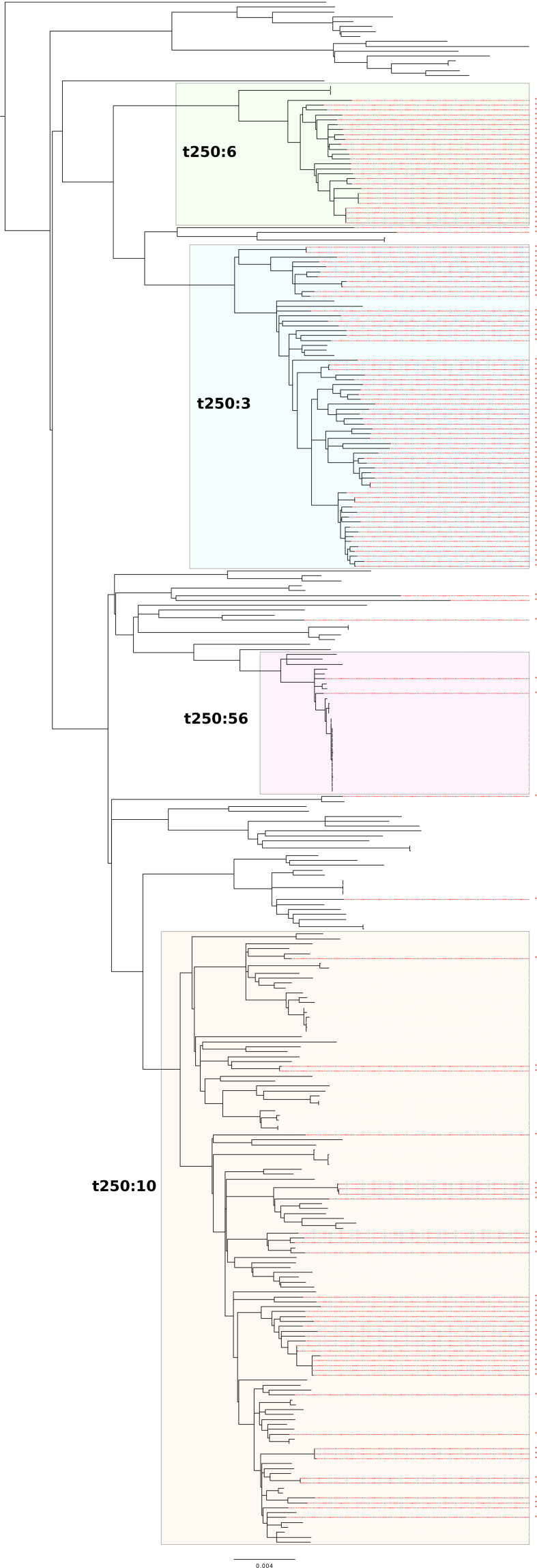
Phylogeny of isolates belonging to ST21C1 from cases residing in England, rooted by ST29C2 strain SRR10357210 (see Supplementary material). The three major domestic clades (t250 single linkage clusters) are shaded. Strains harbouring the *stx2a* encoding phage are highlighted in red. Scale bars represent the number of substitutions per site.

**Fig. 3.**
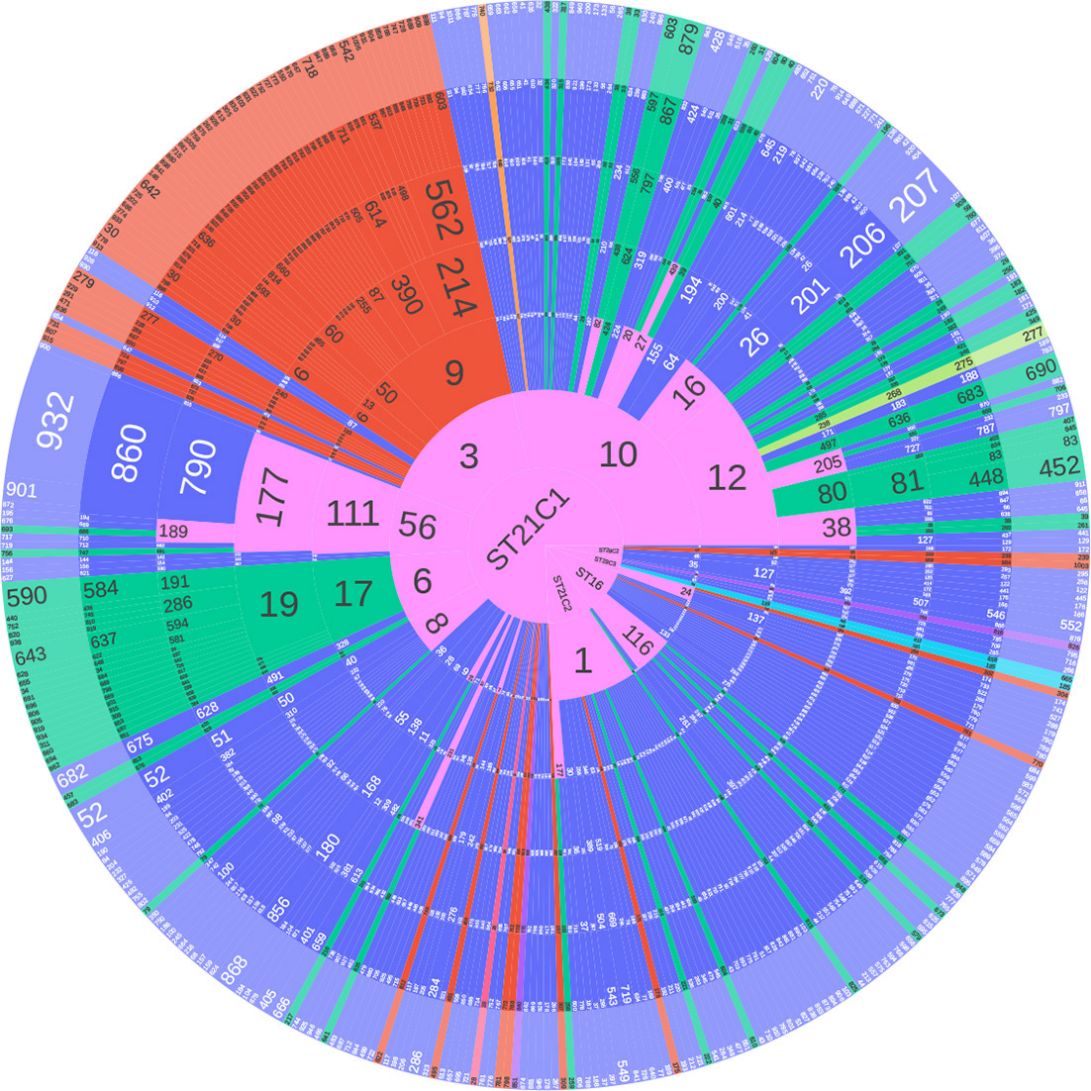
Sunburst diagram showing the distribution of isolates belonging to each lineage and sub-lineage, and six descending concentric circles represent single linkage SNP clusters at the 250-, 100-, 50-, 25-, 10- and 5-SNP levels. The numbers represent the single linkage SNP cluster designation at each level. For example, the SNP type or SNP address for Outbreak A is 56.111.177.790.860.932. The size of each segment represents the proportion of isolates in that cluster. Segments are coloured based on *stx* profile: Blue (*stx1a*), red (*stx2a*), green (*stx1a*, *stx2a*). Pink is where the child segments comprise more than one *stx* profile.

In a previous study, Ogura *et al.* [8] showed that ST29 could be delineated into three clonal complexes (ST29C1–3) and ST21 separated into two large clusters, ST21C1 and ST21C2. Of the 24 ST29 isolates described here 11 (45.8 %) clustered into ST29C2, 13 (54.2 %) into ST29C3 and no ST29C1 isolates were observed (Fig. 1). Ogura *et al*. [8] found that ST29C3 comprised strains of STEC O26:H11 that were previously designated the ‘new European clone’. Of the 13 isolates belonging to ST29C3 in this study, only one had *stx2a* (*stx1a n*=9, *stx1c n*=2, *stx2a n*=1, *stx2c n*=1). ST21C1 contained 314/349 (90.0 %) of the isolates of ST21 in the PHE dataset, of which four isolates were single-locus variants of ST21. There were 40/349 (11.5 %) isolates that belonged to ST21C2, with one isolate with a unique ST. Within ST21C1 there were 38 250-SNP single linkage clusters (t250). The majority of isolates (241/314, 76.8 %) clustered into four 250-SNP clusters which were designated t250 : 10 (*n*=118), t250 : 3 (*n*=65), t250 : 6 (*n*=29) and t250 : 56 (*n*=29). Within t250 : 10 and t250 : 56, 77/118 (65.3 %) and 27/29 (93.1 %) isolates respectively had *stx1a* and 38/118 (32.2 %) and 2/29 (6.9 %) isolates respectively had *stx1a/stx2a*, whereas in t250 : 3 the most common *stx* profile was *stx2a* (60/65, 92.3%) and within t250 : 6 the majority of isolates encoded both *stx1* and *stx2a* (26/29, 89.7 %). From the phylogeny it can be observed that the acquisition of *stx2a* has occurred on multiple occasions, with over ten separate acquisition events in t250 : 10 alone (Fig. 2). The distribution of *stx* profiles within each ST at six different levels of relatedness of single linkage clusters at the 250-, 100-, 50-, 25-, 10- and 5-SNP levels is shown in Fig. 3.

**Fig. 4.**
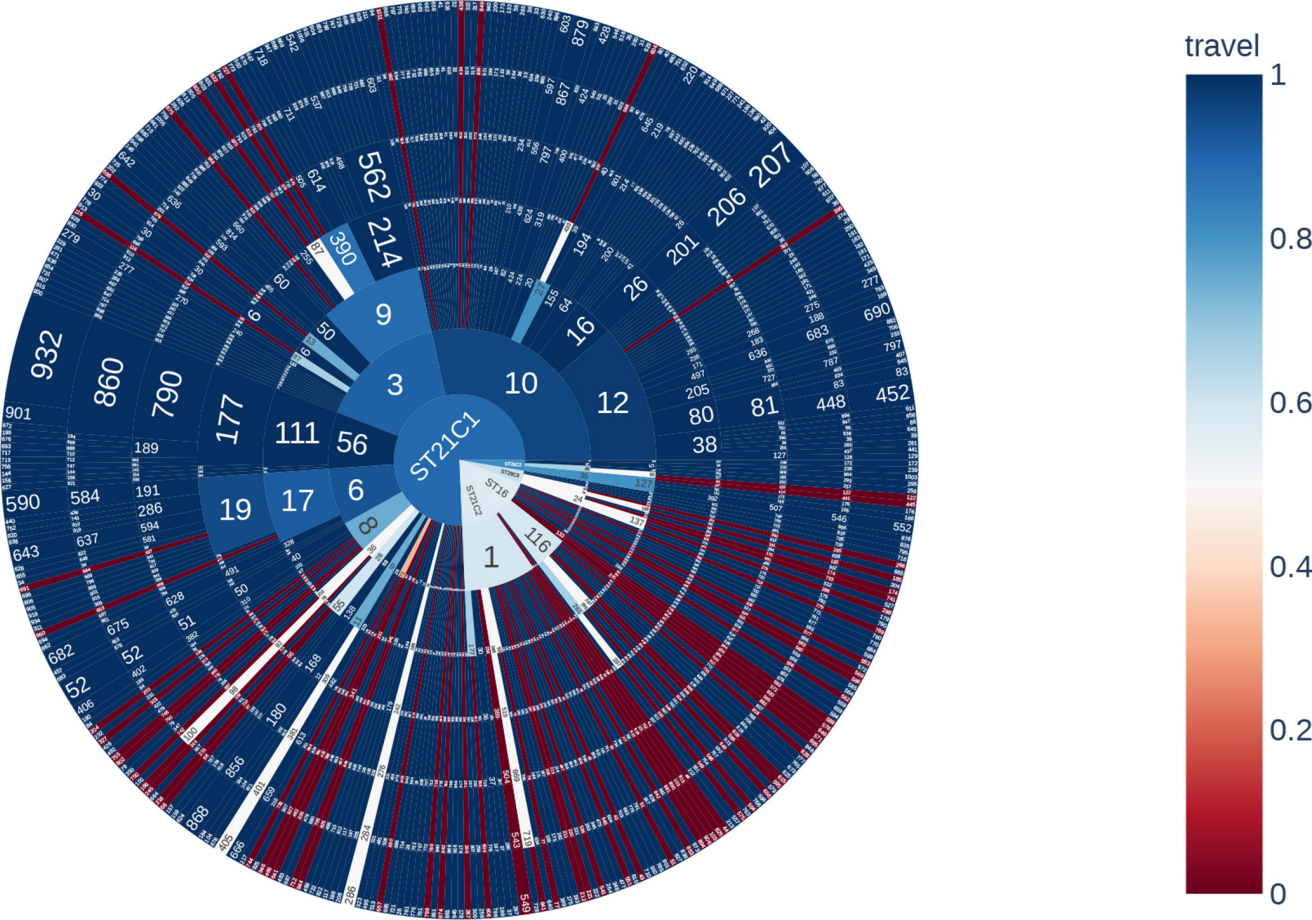
Sunburst diagram showing the distribution of isolates belonging to each lineage and sub-lineage, and six descending concentric circles represent single linkage SNP clusters at the 250-, 100-, 50-, 25-, 10- and 5-SNP levels. The segments were coloured based on the proportion of isolates from cases reporting foreign travel within 7 days of the onset of symptoms, with dark blue being no cases reporting recent travel and dark red being all cases reporting recent travel outside the UK.

In Fig. 4, the segments are coloured based on the proportion of cases that reported foreign travel within 7 days of the onset of symptoms. Within ST21, there was a higher proportion of travel-related cases linked to isolates in t250 : 1 (11/30, 36.6 %) and t250 : 8 (4/16, 25.0 %), compared to isolates in t250 : 3 (6/65, 9.2 %), t250 : 10 (5/118, 4.2 %) and t250 : 6 (2/29, 6.9 %) of which the majority were domestically acquired. The proportions of isolates belong to cases reporting recent travel abroad in ST16 and ST29 were 16/37 (43.2 %) and 7/24 (29.2 %), respectively.

### Analysis of isolates with sequences within the same 5-SNP single linkage community cluster

Of the 416 isolates from individual patients in this study, 330/415 (79.5 %) isolates were identified as belonging to a sporadic case, 67/415 (16.1 %) fell within a 5-SNP single linkage cluster of another isolate, and 18/415 (4.3 %) were from cases belonging to household clusters (Table S1, Fig. 3).

After removing duplicate isolates from the same patient and selecting the earliest representative from the same household, 19 5-SNP single linkage clusters were identified, and designated community clusters. Of these, 6/19 (21.1 %) clusters comprising 14 isolates included cases reporting recent travel to either Egypt, Mexico, Poland, Spain or Sri Lanka (Fig. 4). Cases from one cluster reported travelling abroad but did not specify a destination. For travel-related clusters the median cluster size was two cases (with a maximum of three cases). The median SNP distance between isolates in travel-related cases was 1.5 SNPs (minimum 0 and maximum 3) and the median time interval between cases was 9.5 days, with a minimum of 0 days and maximum of 389 days.

**Fig. 5.**
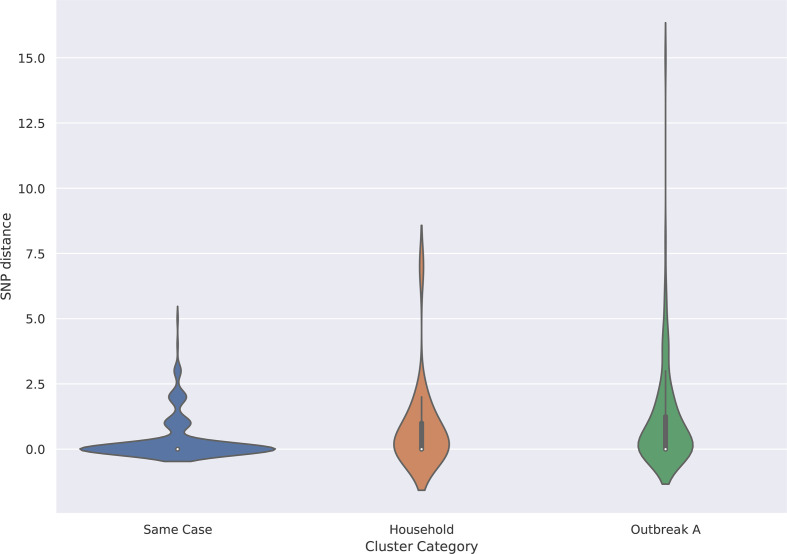
Violin plot showing same case, same household and known outbreak versus SNP distance.

Of the remaining 13 5-SNP community clusters, one cluster comprised 16 isolates of STEC O26:H11 harbouring *stx1a* only (Outbreak A). This outbreak occurred in November 2019 and involved 16 individuals residing in England. Epidemiological analysis identified contaminated salad leaves used as a component of sandwich filling as the most likely vehicle of infection. The median pairwise SNP distances between isolates was 0 SNPs (minimum SNPs=0, maximum SNPs=15) (Fig. 5). The 15 SNPs between two isolates was caused by a recombination event involving an Rha family bacteriophage regulatory protein that contributed 14/15 SNPs. The median time between receipt of isolates was 0 days (minimum days=0, maximum days=34), and residing a median of 206 km (minimum distance=52 km, maximum distance=473 km) from each other respectively. This outbreak cluster fell within a 10-SNP single linkage cluster of three additional cases. These three cases were excluded from the outbreak case definition based on the date of onset of symptoms, and the lack of common exposures. However, although the vehicle and/or transmission route may have been different, it is likely that the strains originated from closely related sources or the same animal reservoir.

There were 35 isolates belonging to the 12 remaining community clusters. The median cluster size was two cases (with a maximum of six cases), the median SNP distance between isolates was 1 SNP (with a maximum of 8 SNPs) and the median time interval between cases was 13 days (0–640).

### Distribution of pairwise distance between isolates from the same case or same household

There were 133 isolates from 46 cases where multiple isolates were sequenced from the same person. Of these 46 cases, 35/46 (76.1 %) cases had two isolates, 3/46 (6.5 %) cases were linked to three isolates, and a further three cases had four isolates. There were five cases with five or more sequential isolates. The median time between receipt of serial isolates was 5 days, with a minimum of 0 days and a maximum of 77 days. The median SNP distance between isolates from the same case was 0 SNPs with a maximum of 5 SNPs (Fig. 5).

There were 18 isolates that were part of seven separate household clusters; 4/8 households had two cases, 2/8 households had three cases and one household had four cases. The median time between receipt of isolates from the same household was 3 days with a minimum of 0 days and a maximum of 52 days. The median SNP distance between isolates from the household was 0 SNPs with a maximum of 7 SNPs (Fig. 5).

## Discussion

Although surveillance for non-O157 STEC is not yet as comprehensive as that in place for STEC O157:H7, the implementation of the GI PCR has improved our understanding of the burden of disease caused by STEC O26:H11 in England. Analysis of the WGS data in this study shows that over the last 5 years, infection in humans has been dominated by four sub-clades, designated 3, 6, 10 and 56, all belonging to ST21. Of these major clades, Clades 3, 6 and 10 appear to be mainly associated with domestically acquired infection, although minor sub-clades with each clade show some evidence of a non-domestic origin, with a higher number of cases reporting travel outside the UK within the incubation period. The majority of isolates in Clade 56 were part of an outbreak.

In contrast to ST21, ST16 and ST29 were isolated less frequently and comprise a relatively high proportion of isolates from cases reporting recent travel, and these STs are less likely to be endemic in the UK. Previous studies have described a highly virulent clone of STEC O26:H11 ST29 with *stx2a*, identified as an emerging cause of HUS in Europe and referred to as the ‘new European clone’ [7, 8]. Although we identified 13 isolates that belonged the ‘new European clone’ group (ST29C3), only one isolate had the *stx* profile that is characteristic of this clone, *stx2a*. Representatives of the more recently described French clone of STEC O26:H11 ST29, characterized by the presence of *stx2d* [13], were not identified among the isolates described in this study.

Of the three major clades in this dataset, Clades 3 and 13 and multiple subsets of Clade 10 have acquired *stx2a-*encoding bacteriophages, and therefore have the potential to cause severe clinical outcomes, including STEC-HUS [40]. Recent studies have also highlighted the role of Stx1a as a marker for severe clinical outcomes, specifically as a cause of bloody diarrhoea and increasing the risk of hospitalization [40, 41]. In England, the STEC Operational Guidance focuses on public health follow up with respect to administering an enhanced surveillance questionnaire (ESQ) and requiring microbiological clearance of patients in risk groups of those cases with STEC harbouring *stx2* (https://www.gov.uk/government/publications/shiga-toxin-producing-escherichia-coli-public-health-management). Consequently, ESQs on a subset of patients infected with STEC O26:H11 harbouring *stx1a* only were not available, thus hindering any future epidemiological analysis of clinical outcomes and exposure risks.

In the UK, outbreaks caused by STEC O26:H11 appear to occur less frequently than outbreaks of STEC O157:H7 [28, 42]. It is uncertain whether this is a true reflection of differences in the burden of disease, animal reservoirs and/or transmission routes, or due to the limitations of the surveillance system for detecting cases of STEC O26:H11. During the 5-year study period, there were 13 5-SNP single linkage clusters, of which only one was linked to a known vehicle of infection, specifically contaminated salad leaves. However, nine of the remaining 12 clusters were temporally related, of which 6/9 occurred in a restricted geographical region, providing circumstantial evidence that outbreaks do occur in England, and can be either local or national. Local outbreaks may be foodborne or have an environmental source, whereas geographically dispersed cases are more likely to be foodborne. Outbreaks of STEC O26:H11 caused by contaminated beef and dairy products indicate that the source and transmission of this serotype are likely to be similar to those of STEC O157:H7 [43–45].

We identified a number of household clusters during this study, and together with others in the literature describing outbreaks in nursery school settings, these data provide evidence that person to person transmission of STEC O26:H11 can occur and that, like STEC O157:H7, the infectious dose is likely to be low [20, 46]. As described in a recent study from an outbreak in Italy, we also identified a number of children who continued to shed STEC O26:H11 for weeks and even months after becoming asymptomatic [21].

As previously reported for STEC O157:H7, in this study isolates from cases with known epidemiological links, specifically those from the same patient, same household or same outbreak with an established source, for the most part fell within 5-SNP single linkage clusters [21]. Further analysis revealed that the SNP differences between one set of isolates from one household and from one isolate belonging to the outbreak fell outside the 5-SNP threshold and were due to phage-mediated recombination events. STEC O26:H11 has an extensive prophage repertoire comprising up to 15 % of the genome [8] and these loci are known to be subject to intra-strain and inter-strain recombination events [47]. As such every effort must be taken to detect and mask these regions of relatedness during the analysis, as the incorporation of exogenous DNA may distort interpretations of genetic similarity.

Our study showed that exposure to foodborne STEC O26:H11 capable of causing severe clinical outcomes, including STEC-HUS, is a risk to public health in England. A single linkage cluster threshold of 5 SNPs has utility for the detection and investigation of both persistent and point source outbreaks of STEC O157:H7 [28, 29], and the WGS data analysed here indicate that this threshold is also appropriate for STEC O26:H11. The lack of comprehensive microbiological and/or epidemiological surveillance of this STEC serotype is a concern. There is a need to expand the implementation of methods capable of detecting STEC O26:H11, specifically PCR targeting *stx* at the local hospital level. Studies focusing on the detection of STEC O157:H7 from food and animal samples should be extended to include STEC O26:H11 to better understand the zoonotic reservoir and transmission routes of this pathogen. Studies investigating the clinical outcomes and common exposures of cases of STEC O26:H11, and comparisons with those reported by cases of STEC O157:H7, are also required.

## Supplementary Data

Supplementary material 1

Supplementary material 2
