# Supplementary figures and images for: Phylogenetic context of Shiga toxin-producing Escherichia coli serotype O26:H11 in England
Source: Microb Genom. 2021 Mar 24;7(6):000551. doi: 10.1099/mgen.0.000551 (PMC8627664; doi:10.1099/mgen.0.000551)

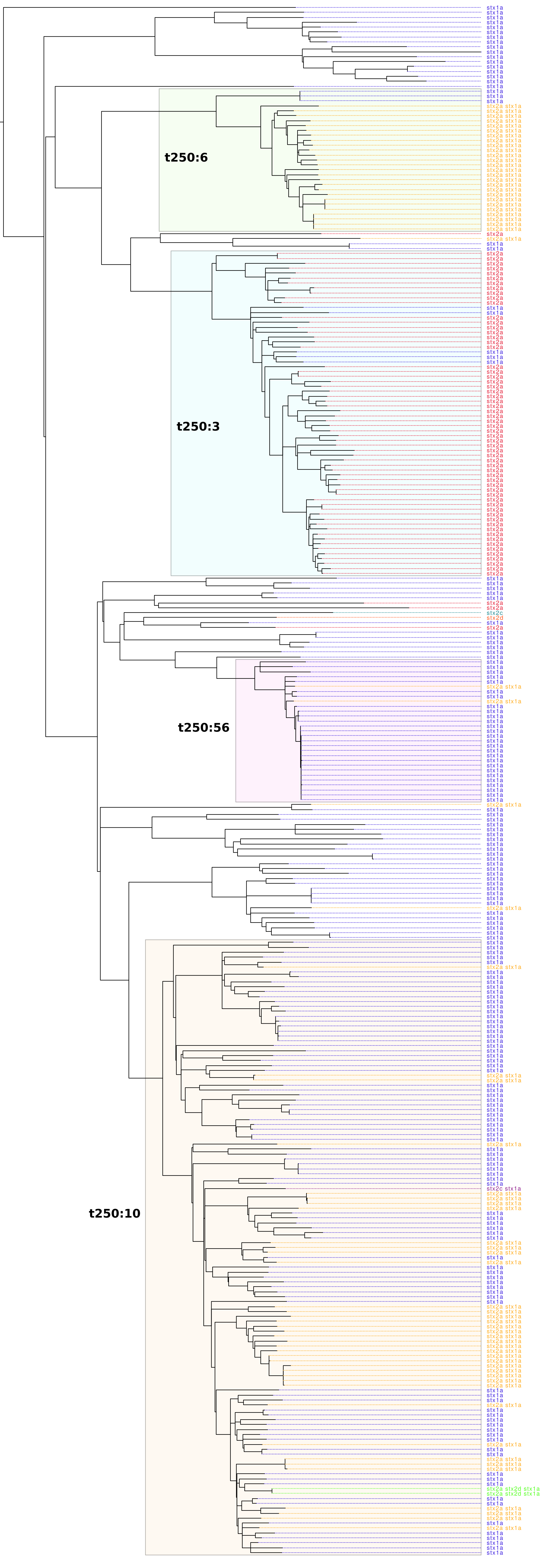

Supplement: Supplementary material 1 [file mgen-7-0551-s001.pdf]
